# Hysteretic device characteristics indicate cardiac contractile state for guiding mechanical circulatory support device use

**DOI:** 10.1186/s40635-021-00426-3

**Published:** 2021-12-20

**Authors:** Brian Y. Chang, Zhengyang Zhang, Kimberly Feng, Noam Josephy, Steven P. Keller, Elazer R. Edelman

**Affiliations:** 1 Institute for Medical Engineering and Science, Massachusetts Institute of Technology, Cambridge, MA USA; 2 Program in Health Sciences and Technology, Harvard Medical School, Boston, MA USA; 3 Abiomed, Danvers, MA USA; 4 Division of Pulmonary and Critical Care Medicine, Brigham and Women’s Hospital, Boston, MA USA; 5 Division of Cardiovascular Medicine, Brigham and Women’s Hospital, Boston, MA USA

**Keywords:** Mechanical circulatory support, Hysteresis, Cardiovascular monitoring, Contractility, Device weaning

## Abstract

**Background:**

Acute heart failure and cardiogenic shock remain highly morbid conditions despite prompt medical therapy in critical care settings. Mechanical circulatory support (MCS) is a promising therapy for these patients, yet remains managed with open-loop control. Continuous measure of cardiac function would support and optimize MCS deployment and weaning. The nature of indwelling MCS provides a platform for attaining this information. This study investigates how hysteresis modeling derived from MCS device signals can be used to assess contractility changes to provide continuous indication of changing cardiac state. Load-dependent MCS devices vary their operation with cardiac state to yield a device–heart hysteretic interaction. Predicting and examining this hysteric relation provides insight into cardiac state and can be separated by cardiac cycle phases. Here, we demonstrate this by predicting hysteresis and using the systolic portion of the hysteresis loop to estimate changes in native contractility. This study quantified this measurement as the enclosed area of the systolic portion of the hysteresis loop and correlated it with other widely accepted contractility metrics in animal studies (*n* = 4) using acute interventions that alter inotropy, including a heart failure model. Clinical validation was performed in patients (*n* = 8) undergoing Impella support.

**Results:**

Hysteresis is well estimated from device signals alone (*r* = 0.92, limits of agreement: − 0.18 to 0.18). Quantified systolic area was well correlated in animal studies with end-systolic pressure–volume relationship (*r* = 0.84), preload recruitable stroke work index (*r* = 0.77), and maximum slope of left ventricular pressure (d*P*/d*t*_max_) (*r* = 0.95) across a range of inotropic conditions. Comparable results were seen in patients with d*P*/d*t*_max_ (*r* = 0.88). Diagnostic capability from ROC analysis yielded AUC measurements of 0.92 and 0.90 in animal and patients, respectively.

**Conclusions:**

Mechanical circulatory support hysteretic behavior can be well modeled using device signals and used to estimate contractility changes. Contractility estimate is correlated with other accepted metrics, captures temporal trends that elucidate changing cardiac state, and is able to accurately indicate changes in inotropy. Inherently available during MCS deployment, this measure will guide titration and inform need for further intervention.

## Background

Ischemic disease is the leading cause of acute heart failure and cardiogenic shock, life-threatening conditions where impaired contractility and inadequate cardiac output lead to hemodynamic instability and systemic hypoperfusion [1, 2]. Prompt coronary intervention reduces the likelihood of subsequent complications following ischemic insult; however, further reduction in time to revascularization has not yielded benefit [3]. Restoring hemodynamic stability has become a mainstay therapy, however mortality rates remain as high as 40% with traditional medical therapy [4].

Mechanical circulatory support (MCS) devices offer a therapeutic modality where mechanical pumps restore hemodynamic stability for patients in heart failure [5, 6]. Percutaneous forms of MCS devices can provide additional benefits by directly unloading the ventricle [6, 7]. By also reducing wall stress and metabolic demands of the ischemic ventricle, these devices may lead to improved outcomes [8–10], and promote recovery of endogenous cardiac function to rescue patients in acute heart failure [10, 11]. However, one of the obstacles to successful widespread use of percutaneous MCS devices is the difficulty in titrating for an appropriate degree of support, for which accurate assessment of underlying cardiac state is critical [10, 11]. Frequent assessment for contractility improvement is an essential aid for intensivist management of MCS devices, which includes weaning, to optimize outcomes [11, 12]. However, intermittent surrogate measures, such as ultrasound imaging and invasive catheter measurements, are often the only available options for clinicians. Providing a novel continuous measure of cardiac contractility may thus improve support of patients in acute heart failure and cardiogenic shock while being supported by MCS.

Mechanical circulatory support devices themselves present a platform to determine cardiac state in unique ways without additional intervention. Device signals can vary with patient state, especially in the setting of residual cardiac function [13]. While devices are quantified by manufacturers with static relationships, they yield dynamic phenomenon that deviate from static relationships when used in conjunction with any residual cardiac pulsatility. We and others have previously demonstrated that this dynamic signal can reflect various physiologic measures, including left ventricular end-diastolic pressure and cardiac output [14–17]. These investigations elucidated a device-heart interaction in the form of a hysteretic relationship based off of a modified pump-performance curve that varies with contractility [15]. Traditionally, hysteresis complicates device performance characterization due to non-linear relationships. However, the degree of hysteresis can reflect characteristics of the cardiac cycle when estimated using models.

To demonstrate the utility of hysteresis, we propose that the portion related to contraction may reflect underlying cardiac contractility. Here, we present an approach to model and estimate MCS device-heart hysteresis and demonstrate its utility through quantification that can estimate changing cardiac contractile state. While device signals have been previously used to estimate contractility, they were not done so in the context of hysteretic interaction with the heart using percutaneous devices [18, 19]. We show that the portion of the hysteresis loop that corresponds to cardiac isovolumetric contraction can be modeled to calculate an enclosed area without measuring intracardiac pressure. The enclosed area correlates closely with other measures of contractility. Significantly, this measure is calculated using only device signals, does not require device modification, and can indicate changing contractile state of the heart without additional intervention. We evaluated hysteresis modeling and the subsequent contractility estimation in a series of animal models using acute interventions to induce a range of contractile states and leveraged available diagnostic instruments for comparison to several contractility measures. Clinical validation was completed using invasive hemodynamic measures from a series of patients undergoing percutaneous coronary intervention (PCI).

Device-derived hysteresis estimation provides insight into cardiac state without additional intervention or need to measure intracardiac pressures during MCS support. Quantification of the systolic portion of hysteresis specifically allows a novel, continuous, and reliable measure of cardiac contractility during the use of MCS. Such a contractility marker will be crucial for determining and optimizing the appropriate degree of support, indicating the need for further intervention, and developing future closed-loop control of MCS devices.

## Methods

These methods aim to demonstrate the utility of device-heart hysteresis modeling from a percutaneous mechanical circulatory support device. Contractility is specifically estimated using hysteresis modeling and compared with other common measures of contractility in acute animal models and patients undergoing PCI.

### Impella and device–heart interaction induced hysteresis

**Fig. 1:**
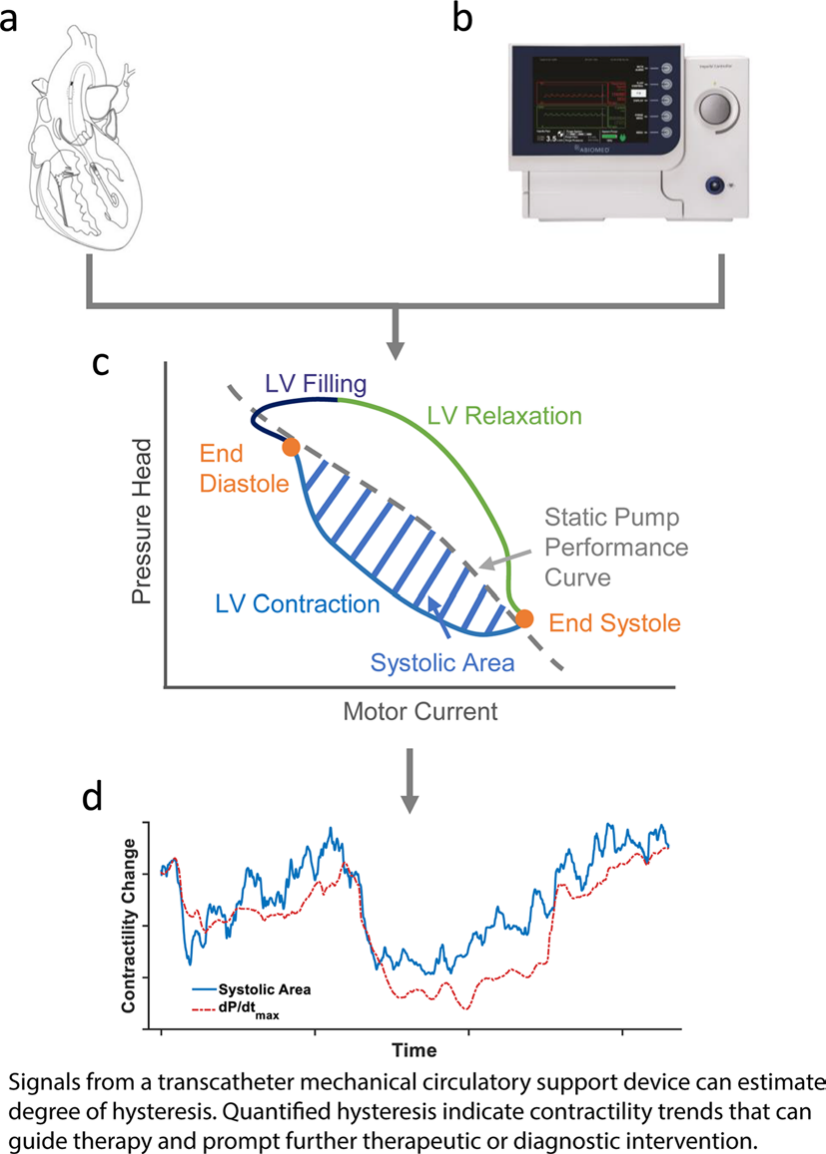
Origins of device–heart interactions and hysteresis quantification as a contractility metric. **a** The Impella is an indwelling percutaneous mechanical circulatory support device that is connected to a controller. The device crosses the aortic valve with an inlet in the left ventricle and an outlet in the aorta. The device maintains a constant rotational speed to provide constant flow out of the ventricle. **b** The Impella controller records device signals (motor current, motor speed) that can be used to continuously derive device–heart relationships. **c** Plotting pressure head across the device with motor current shows that residual cardiac pulsatility induces a hysteretic relationship in device signals that varies with contractility. This hysteresis can be estimated by using the device signals alone without direct measurement of pressure head. Separated by cardiac phase, the systolic portion of the hysteresis loop can be quantified as a measure of contractility. **d** Quantifying the systolic portion of this relationship yields a continuous marker that can indicate changes in native contractility. This marker can be used to indicate increases or decreases in contractility to prompt further therapeutic or diagnostic intervention

The Impella (Abiomed, Danvers, MA) percutaneous MCS device was used as the paradigmatic device in this study due to its availability, mode of operation, and clinical indication. This catheter-mounted mixed-flow pump bridges the aortic valve to unload the left ventricle in the setting of impaired function (Fig. 1A). Blood is drawn from an inlet port residing within the ventricle by a rotating impeller that sits within the aorta. Blood moves antegrade to provide systemic perfusion in conjunction with remaining cardiac pulsatility. The impeller rotates at a clinician-determined fixed speed that typically ranges from 23,000 to 46,000 rotations-per-minute (P-1 to P-8) maintained by modulating motor current. The Impella is placed percutaneously and connected externally to a controller that allows clinician control and records device signals: motor current, motor speed, and placement signal, which corresponds to aortic pressure (Fig. 1B). These signals can be used to calculate patient parameters in real-time or retrospectively [14–16].

As a mixed-flow pump, the amount of blood flow through the pump depends on both the pump rotational speed and the pressure head, the difference between aortic pressure and left ventricular pressure (LVP). This relationship is characterized by a performance curve that relates flow rate through the pump with the pressure head at a given rotor speed. A modified performance curve can be generated using the directly measured motor current signal, a measure of pump power and a surrogate of load experienced by the pump, to show its inverse curvilinear relationship with pressure head under steady and non-pulsatile flow conditions (Fig. 1C) [15, 20]. In the presence of a pulsatile heart, the pump’s operating point oscillates between peak ejection and filling through contraction and relaxations. However, the movement between these two points is directionally dependent, resulting in a hysteretic loop. Hysteresis is broadly defined as a phenomenon with measurable outputs that are dependent on their prior states due to an inherent delay to the input, in this case pressure differential and motor current. Thermodynamically, the finite time required to move between points results in an entropic driver for hysteresis that resembles that seen with any viscoelastic phenomenon [21, 22]. This is notably distinct from cyclic behavior or loops that are a result of a change in the physical environment such as operation of valves. Instead, hysteresis manifests as a loop that deviates from its static performance curve (Fig. 1C), with the degree of deviation reflective of the dynamic driver that determines the shift between peak ejection and relaxation states. Consequently, the degree of hysteresis is not a fixed quantity but a reflection of cardiac behavior. The characteristics of how the heart contracts and relaxes are reflected in the degree of hysteresis and observed when the modified pump performance curve is measured. Changes in hysteresis with consistent device function are a reliable reflection of changes in cardiac state. Therefore, quantitative evaluation of the pressure head–motor current hysteresis can provide insight into the cardiac state.

### Systolic area and contractility measures

The modified pump performance curve hysteresis loop reflects the cardiac cycle and can be sequentially separated into cardiac phases of relaxation, filling, and contraction in a counter-clockwise direction, with the points corresponding to end-diastole and end-systole being readily identifiable (Fig. 1C). Our prior work had first described this hysteresis loop and demonstrated stability specifically at end-diastole across various contractile states, allowing for estimation of left ventricular end-diastolic pressure by predicting the pressure head from motor current [15]. Conversely, the remaining portion of the hysteresis loop can vary dramatically with changing contractility [15]. Contractility, an inherent property of the heart that is independent of loading conditions, drives the size and shape of hysteresis during the contraction phase of the hysteresis loop (Fig. 1C). This effect can be quantified by measuring the enclosed area of the contractile phase of the hysteresis loop using a numerical trapezoidal Riemann sum (Fig. 1C). However, this requires ability to directly measure the pressure head, which is not readily available without additional intervention. Therefore, the ability to estimate pressure head is crucial to utilize any hysteresis characteristics to indicate changing state.

The viscoelastic behavior of blood flow and the pump can be directly used to estimate pressure head without using intracardiac pressure measurement. Hysteretic behavior is not unique to motor current measurements and also exists in traditional pump performance curves. Modifying existing approximations for pump hysteresis that are based off of the Euler fluid equations, which relate fluid pressure gradient with flow velocity and flow acceleration, can yield an estimation for pressure head without need for additional measurement of intracardiac pressure [23]. Motor current is related to the volumetric flow through the device such that flow rate and acceleration can be represented using the motor current waveform. With the addition of an empirically determined motor speed term, motor current and the slope of the motor current waveform can be used to directly estimate pressure head as seen below:

$$\Delta P = a*i + b*\frac{{{\text{d}}i}}{{{\text{d}}t}} + c\omega^{2} .$$

The pressure head across the device ($\Delta P$) is a function of the motor current (*i*), the motor current’s rate of change $\left( {\frac{{{\text{d}}i}}{{{\text{d}}t}}} \right)$, and the motor speed ($\omega$). Each term is preceded by a device-specific coefficient (a, b, c). Accuracy can be improved by identifying phase-specific coefficients due to the differing characteristics within each cardiac phase (Fig. 1C). The coefficients corresponding to the systolic area are found by gating to features in the motor current waveform to identify end-diastole and peak-systole [24]. Hysteresis modeling coefficients (a, b, c) can be determined upon mock circulatory loop pre-characterization [15], and were derived from baseline conditions in this study and for use across all cardiac states.

Pressure head from the above equation was used to estimate hysteresis without pressure measurement. The accuracy of this hysteresis model was evaluated through comparison with directly measured hysteresis in patient and animal data. The estimated hysteresis was then quantified using the enclosed systolic area as a measure of changing contractility. To track changes in contractility, values are normalized to baseline states to represent relative changes in state and allow pooled analysis across animals and patients. This systolic area calculation can be performed beat-to-beat in real-time to track and detect changes in contractility that would otherwise not be available without additional invasive diagnostic tools. As a new MCS device-derived measure of contractility, this approach was compared with several other commonly accepted contractility metrics.

### Continuous contractility measures

Cardiac contractility is a systolic property of the heart that can be quantified using the end-systolic pressure–volume relationship (ESPVR) and preload recruitable stroke work index (PRSWi) with reduced sensitivity to loading conditions [25, 26]. These parameters can be directly measured using pressure–volume loops at varying loading conditions during MCS [27]. However, due to clinical and practical barriers to altering loading conditions and continuously measuring ventricular volume in patients, surrogate measures are more often used [28]. While these indirect measures provide insight into patient status, they can only be inferred from loading state and require additional intervention [29].

Classically, contractility is described as increasing ventricular elastance that is characterized by the peak elastance at end-systole, or ESPVR. Visualized in a pressure–volume diagram, ESPVR is displayed as a line connecting the point of end-systole with a hypothetical volume intercept (*V*_0_) that remains relatively constant. Contractility is then estimated using the slope from pressure–volume measurements [25]. However, there are restrictions to this approximation. ESPVR is often non-linear, concave or convex, limiting direct comparison between linear approximations and providing inconsistent approximation of the hypothetical *V*_0_ [25]. In addition, practical challenges add additional error. This is especially evident with interventions that alter loading conditions. These must be used to induce changes to measure ESPVR and *V*_0_ and can produce conflicting results for the same contractile state [30].

Difficulty in consistently measuring ESPVR values and its sensitivity to afterload has led to increased interest in PRSWi as a more stable and robust measure of contractility [26, 30]. PRSWi is derived from the linear relationship between left ventricular stroke work, the time integral of pressure and flow over the cardiac cycle, and end-diastolic volume [26]. Because PRSWi takes the entire cardiac cycle into account, it may be a more stable measurement than ESPVR [30, 31]. However, ESPVR and PRSWi measurements both require continuous and simultaneous measurements of ventricular pressure and volume as well as an intervention to vary loading conditions over a wide range, making both impractical to obtain regularly in a clinical setting [30]. Limitations in clinically available tools have led to the use of other measures to assess contractility. Echocardiography and other forms of cardiac imaging are frequently used, but can only be performed intermittently and are highly operator dependent [32, 33]. For more continuous assessment, hemodynamic measurement of the maximum LVP slope (d*P*/d*t*_max_) is used when additional intervention is available [28].

There are many contractility metrics that each have limitations in their utility and availability in the clinic. To best represent the context of the results, we compared all three measures with our new measure of contractility derived from MCS device parameters alone.

### Animal models

A series of four acute animal models (~ 75 kg young adult male Yorkshire swine) were used to assess hysteresis modeling and contractility quantification across a range of inotropic conditions induced by pharmaceutical and mechanical interventions. All animals were maintained in accordance with National Institutes of Health (NIH) and Association for Assessment and Accreditation of Laboratory Animal Care (AAALAC) guidelines (CBSET, Lexington, MA); body temperature, oxygen saturation, and electrocardiogram were continuously monitored for the duration of the studies. Anesthesia was induced via intramuscular injection of telazol (6 mg/kg) and maintained using inhaled isoflurane after intubation. An Impella CP was placed into the left ventricle via the right femoral artery. A pressure–volume catheter (Millar, Houston, TX), placed via the left carotid artery, measured continuous LVP and volume. Appropriate placement and aortic valve competency were monitored via fluoroscopy.

Pharmaceutical interventions delivered via the femoral venous sheath induced changes in contractility, and mechanical interventions allowed assessment and induction of an acute heart failure model. Each condition was assessed at two Impella speeds (P-3 and P-6), which are commonly used speed settings that avoid suction from high flow rates and retrograde flow during diastole. A preload reduction to alter loading condition was induced by mechanical occlusion of the inferior vena cava by a balloon-tipped catheter introduced via the right femoral vein. Ventricular pressure–volume data from these points were extrapolated to determine V_0_ values to calculate ESPVR in subsequent interventions. Boluses were given for pharmaceutically induced positive inotropic (epinephrine at 1 µg/kg), and negative inotropic (esmolol at 1.8 mg/kg) conditions. Local ischemia was induced by injecting compressible 45–105 µm diameter microspheres (Hydropearl, Terumo, Tokyo, Japan) into the left main coronary artery through a Judkins left catheter placed via the left femoral artery. Serial boluses of 0.25 mL of microspheres mixed with 10 mL of isotonic saline and 10 mL of contrast were serially injected until LVEDP rose to > 20 mmHg or there was evidence of ventricular decoupling [34].

A research-grade data acquisition system (ADInstruments, Dunedin, New Zealand) continuously recorded a single-lead electrocardiogram and the Millar pressure–volume catheter. Impella signals were recorded on a time-synced controller and retrieved after each trial. LVP signals are used as the reference to evaluate hysteresis estimation and are not part of the systolic area calculation. All calculations of contractility parameters were performed retrospectively.

### Retrospective patient data

Anonymized data were obtained for eight adult patients undergoing elective Impella-supported high-risk PCI due to the availability of hemodynamic measures. Data were obtained with informed consent and with approvals from an Ethics Review Committee and relevant Institutional Review Boards. In total there were two female and six male patients ranging from 49 to 69 years of age. All patients were either NYHA Class II or III heart failure and hemodynamically stable prior to the procedure. Impella device signals were recorded on the controller through the duration of the intervention with support provided at a range of speeds (P-2, P-4, P-6, P-8). Continuous digital LVP tracings, used as a reference signal, were also available from an indwelling pigtail catheter for the duration of the trial. Following procedures, device signals and continuous left ventricular tracings were downloaded and registered for analysis. Hysteresis estimation was evaluated using the indwelling left ventricular tracing as a reference signal to evaluate performance. Changes in contractility were also calculated using dP/dt_max_ from the LVP tracing as a reference. There were no recorded adverse events for any of the eight patients. All calculations of contractility parameters were performed retrospectively.

### Statistical analysis

Hysteresis estimation in animals and patients were evaluated using a two-sided paired *t*-test and visualized using correlation and Bland–Altman plots with repeated measures. Correlation coefficients from regression analysis were used to compare each combination of contractility measures for the pooled animal results. These changes are shown normalized to a baseline state to account for differences in scale between measures and calibration. Prescriptive performance to indicate changes in contractility as determined against moving average changepoint detection in d*P*/d*t*_max_ were performed for each contractility measure using receiver-operator characteristics (ROC) curves and quantified using the area under the curve (AUC). Differences between individual animals and patients are assessed using one-way ANOVA and the Fisher *z*-transformation of the correlation coefficient for each combination to support data pooling. All methods used standard confidence intervals (95%) and significance values (*p* < 0.05). Data and statistical analyses were performed in MATLAB (Mathworks, Natick, MA).

## Results

Hysteresis systolic area can be modeled and calculated using device-only parameters as a marker for tracking changes in underlying cardiac contractility. We evaluated its performance in a series of four acute animal models and eight patients.

### Physiologic characteristics

The four acute animal models allowed the use of invasive diagnostic tools for measurement of ESPVR, PRWSi, and d*P*/d*t*_max_ for comparison with systolic area at 113 distinct points. These points spanned a range of conditions, including microbead-induced acute heart failure with ESPVR from 0.44 to 1.80 mmHg/mL, PRSWi from 10.6 to 76.5 mmHg, and d*P*/d*t*_max_ from 653 to 2731 mmHg/s. Anonymized data from eight patients validated the hysteresis estimation and the systolic area at 10,940 points. Patients displayed varying states with d*P*/d*t*_max_ ranging from 315.5 to 3300.1 mmHg/s.

### Hysteresis estimation

**Fig. 2:**
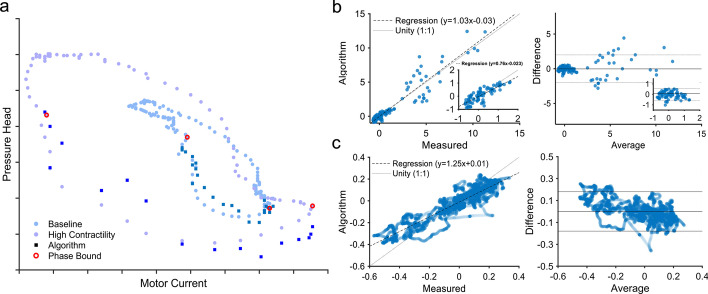
Hysteresis estimation across contractile states. Hysteresis can reflect changes in cardiac state and can be estimated using an algorithm employing device signals alone. **a** Representative animal data show how hysteresis can increase with contractility from a baseline (blue) to a high inotropy (purple). Instead of direct measurement (circles), hysteresis can be accurately estimated at each point during the systolic phase using a motor current relation derived from Euler’s fluid equations (squares). **b** Correlation and Bland–Altman analysis are performed to compare quantification of the systolic phase from direct measurement and estimated hysteresis. The results from hysteresis estimation are well-matched to direct measurement in animals undergoing various acute interventions, with improved limits of agreement when considering ranges typical for cardiogenic shock. **c** A similar comparison is performed using data from patients undergoing percutaneous coronary intervention with results that are consistent with animal results. Notably, patients had more varied baselines, but individually less inotropic variation compared to animal studies with acute interventions

The hysteresis estimation from pressure head calculation was compared with reference direct measurement (Fig. 2A) and yielded results for systolic area that did not differ significantly (*p* > 0.82). Correlation with direct measurement was strong (*r* = 0.94, *p* < 0.05) with a mean bias of − 0.02 and limits of agreement from − 2.0 to 2.0 (Fig. 2B). Within the expected physiologic range for patients supported on MCS, correlation remains comparable (*r* = 0.84, *p* < 0.05) with improved limits of agreement (− 0.57 to 0.7) (Fig. 2B). Patient data also yielded strong results with systolic areas that are correlated (*r* = 0.92, *p* < 0.001) and in close agreement with reference direct measurement (mean bias = 0.002, limits of agreement: − 0.18 to 0.18) (Fig. 2C). These results indicate that hysteresis estimation is a good model for the actual hysteresis phenomenon.

### Hysteresis-based contractility

**Fig. 3:**
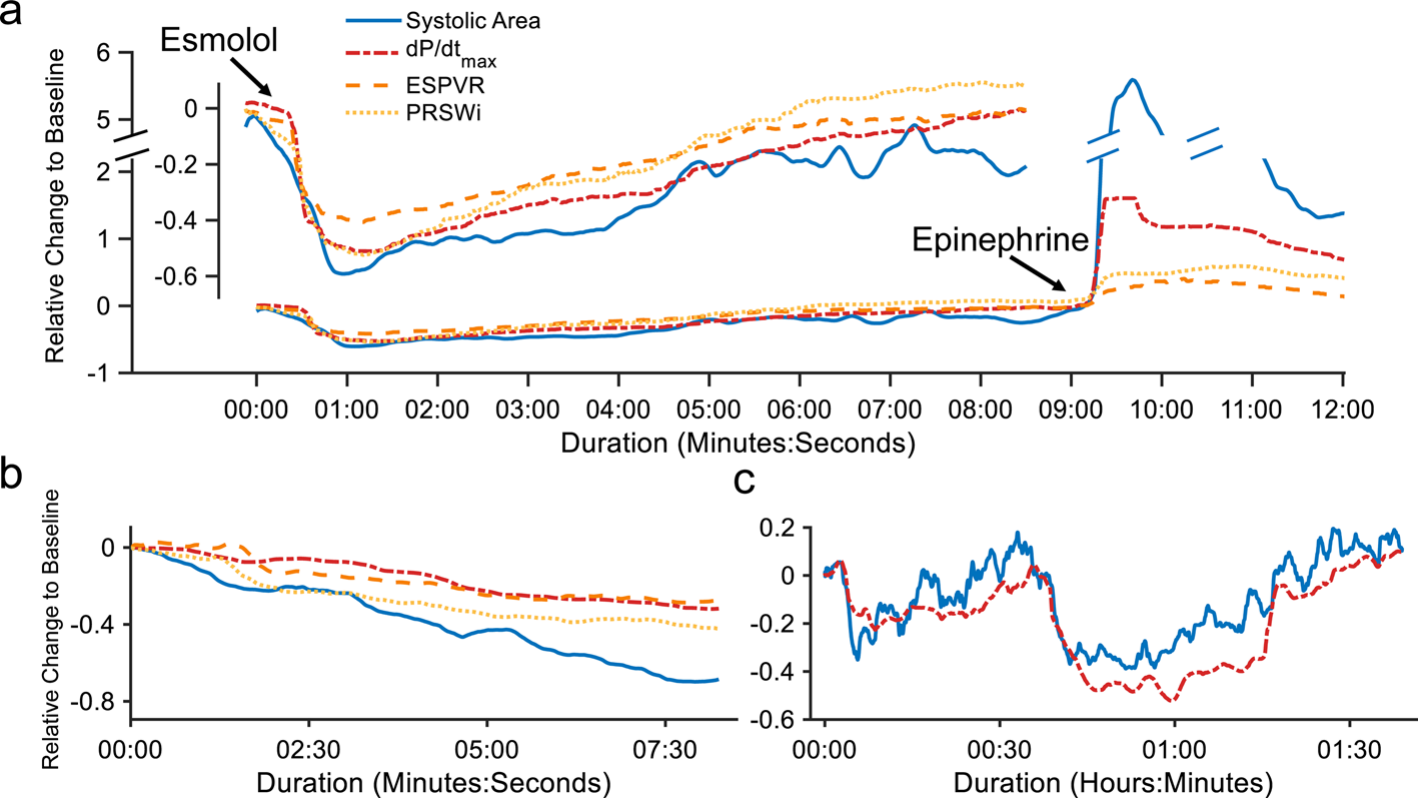
Representative time-courses for contractility metrics. Representative time-series of results in animals and patients show how the different contractility measures vary across interventions. **a** From an acute animal study, a representative time-course shows changes in contractility induced by pharmacologic intervention with the hysteresis-derived systolic area (blue) following trends seen in other reference measurements d*P*/d*t*_max_ (red), ESPVR (orange), and PRSWi (yellow). The inset shows an early negative inotropic response from a bolus of esmolol while the later bolus of epinephrine shows a positive inotropic response. **b** From an acute animal study, the more gradual negative inotropic trend from a serially injected microbead-induced heart failure model is similarly tracked in the systolic area and reference measurements. **c** An example patient time-course during an elective PCI shows inotropic variability through the course of the procedure as indicated by reference d*P*/d*t*_max_ (red) measurement. This variability and trends are similarly represented with systolic area (blue) using values measured clinically from device signals without modification

**Fig. 4:**
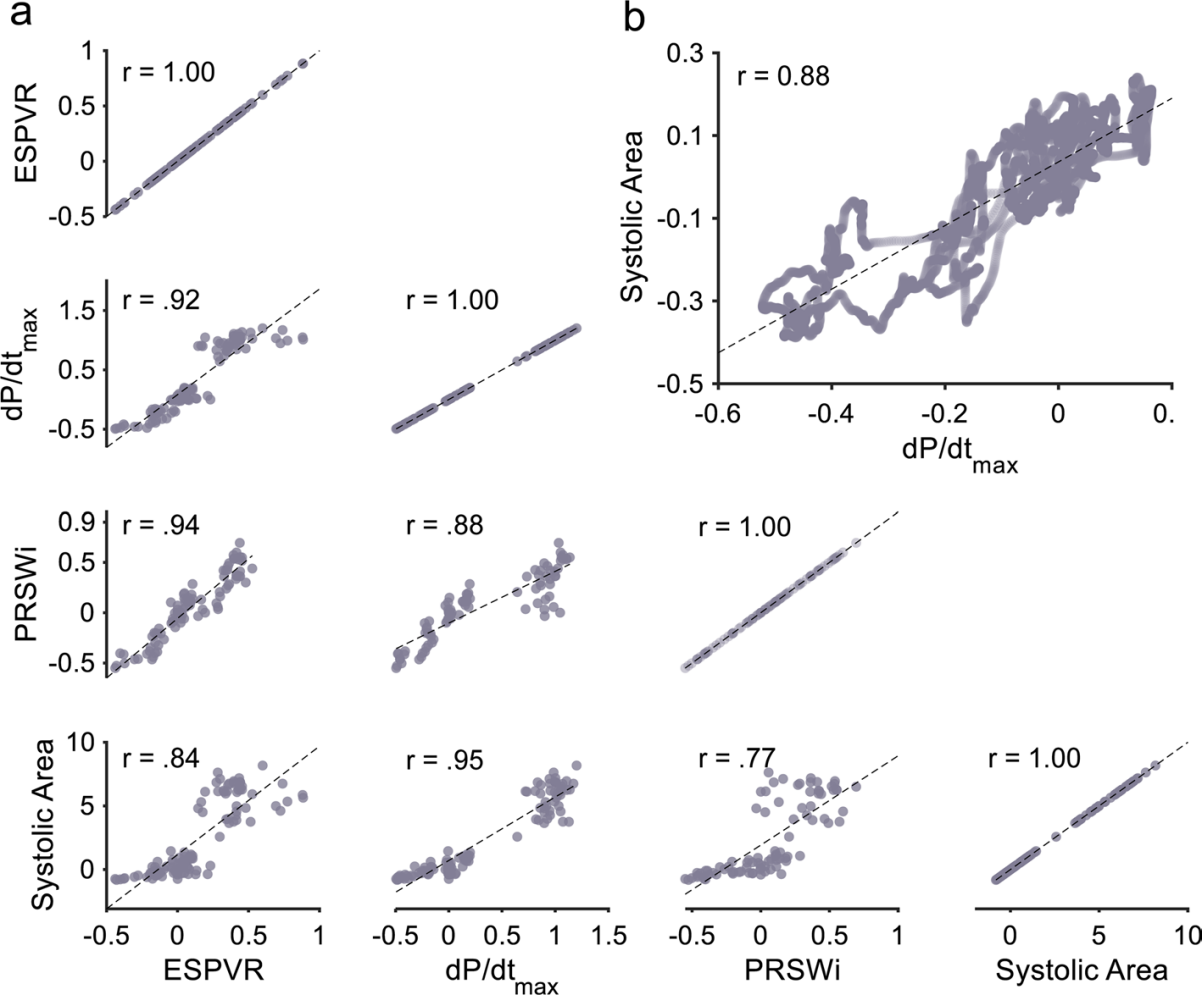
Correlation between contractility measures. Correlation analysis was performed on different combination of contractility measures in animals and patients. **a** All combinations of contractility measures in animals are compared using a matrix of correlation plots without non-significant variation between comparisons. For reference, the unity relationship for each metric is shown on the right most diagonal. **b** The correlation between systolic area and d*P*/d*t*_max_ in patients are also strong and consistent with animal results. Only d*P*/d*t*_max_ was available as a reference measurement in patient data analysis yielding this single correlation

Quantification of the degree of hysteresis is evaluated as a surrogate for changing contractility in the form of systolic area. In a representative animal time-course, the normalized changes in contractility corresponded with each intervention at baseline and correlated to other contractility measures (Fig. 3A). The systolic area also tracked the progression of microbead-induced cardiogenic shock (Fig. 3B). Across all animals, the hysteresis systolic area strongly correlated with ESPVR (*r* = 0.84, *p* < 0.001) and d*P*/d*t*_max_ (*r* = 0.95, *p* < 0.001) with a slightly weaker correlation to PRSWi (*r* = 0.77, *p* < 0.001) (Fig. 4a). There were no significant differences between correlations when considering the confidence intervals from the Fisher *z*-transformation.

A representative patient time-course with the greatest variability showed changes in systolic area that closely correlate with those seen with d*P*/d*t*_max_ (Fig. 3C). When pooled, the systolic area was highly correlated (*r* = 0.88, *p* < 0.001) and comparable with the results seen in the animal models (Fig. 4B).

### Diagnosing contractility changes

**Fig. 5:**
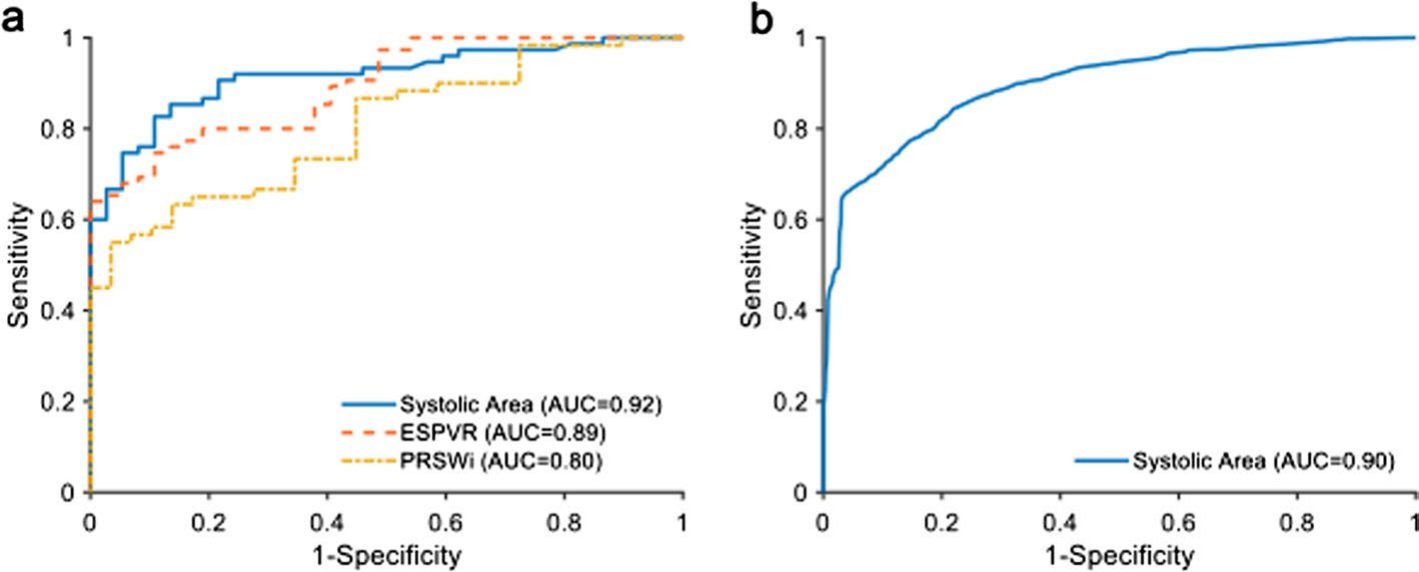
*Receiver operating characteristic curve analysis for diagnostic performance.* Diagnostic performance for prediction of directional change in contractility was quantified using AUC of ROC curves. Each contractility measure was compared with contractility change as indicated by changes in d*P*/d*t*_max_ detected using changepoint detection. **a** In the acute animal models, use of invasive pressure–volume measurement catheters allowed measurement of ESPVR and PRSWi as benchmarks to compare with systolic area performance. Quantified by AUC, systolic area had comparable or superior diagnostic capability to indicate changes in contractility compared to d*P*/d*t*_max_. **b** In patient data, systolic area was compared with AUC and yielded

Efficacy of systolic area to indicate changes in contractility against d*P*/d*t*_max_ was quantified in animal and patient datasets using ROC curve-derived AUC (Fig. 5). ESPVR against d*P*/d*t*_max_ and PRSWi against d*P*/d*t*_max_ were also evaluated in the animal datasets to be used as a benchmark (Fig. 5A). For systolic area, the overall AUC was comparable between animals (Fig. 5A) and patients (Fig. 5B) with 0.92 [0.90 to 0.94] and 0.90 [0.89 to 0.91], respectively. The AUC was 0.99 [0.98 to 1.00] for increasing contractility and 0.95 [0.90 to 0.99] for decreasing contractility in animal data, and 0.89 [0.88 to 0.90] for increasing contractility and 0.97 [0.96 to 0.98] for decreasing contractility in patient data. These are compared with the ESPVR AUC of 0.89 [0.82 to 0.97] and PRSWi AUC of 0.80 [0.70 to 0.90].

## Discussion

MCS devices offer a promising therapy for patients in acute heart failure with potential to support the recovery of endogenous cardiac function [8–11]. However, appropriate use of these devices is dependent on an accurate assessment of cardiac contractility. In particular, weaning the device in the intensive care unit relies on accurate markers of cardiac contractility [10–12]. Despite advances in imaging [32, 33], continuous measurement is needed for monitoring and providing fine-tuned control of MCS devices, and may be the missing link to widespread clinical benefit in treating cardiogenic shock.

Here, we present a novel MCS device-derived contractility metric that utilizes device-heart hysteretic interactions to estimate quantitative trends in contractility with a modified pump-performance curve. A cardiac phase-specific characterization is used to estimate hysteresis during cardiac contraction without need to measure intracardiac pressure. The enclosed area of this portion of hysteresis is quantified as a measure of contractility. Relative changes of this enclosed area track with contractility and correlate well with other commonly accepted metrics and can be used to indicate when contractility changes occur. Since this approach only relies on existing measures from the MCS device, this can be used as an onboard indicator of changing contractility to guide treatment or further diagnostic intervention.

### Hysteresis estimation

Greater contractility increases the degree of observed hysteresis (Fig. 2A). With the Impella, we estimate the degree of hysteresis without intracardiac pressure measurement. The intracardiac measurements performed in this study were only used as a reference signal to compare with algorithm-based estimations. The systolic portion of hysteresis, as bound by end-diastole and peak-systole, was modeled using motor current and speed with a set of coefficients (Fig. 2A). Coefficients from each animal and patient varied slightly and demonstrated feasibility of manufacturing pre-characterization methods in-line with existing flow calibration. The subsequent systolic area was normalized to the values at the beginning of recording track relative changes.

Overall, the modeled systolic area was well correlated with direct measurement and is subsequently representative of systolic area as a contractility measure (Fig. 2). There were no differences in performance between different animals and only a small effect size difference between patients. Metric performance was best within the contractility range that is expected in heart failure (Fig. 2B). Significant deviations were only observed with supraphysiologic contractile states achieved with infusion of positive inotropes in animals (> 2 × baseline) (Fig. 2B)—inotropic states that are unlikely to occur during support of patients in acute heart failure. While animals had relatively similar baselines with wide variation from interventions, patients had widely varied baselines with smaller variations during PCI. Improved limits of agreement for the patient data are likely from reduced variability, though patient states did vary during procedures (Fig. 3C).

### Contractility tracking

Though theoretical descriptions of intrinsic contractility are well accepted, their clinical utility is limited due to need for high-fidelity volume measurement and difficulty in inducing pure loading condition variations within intact physiology [30]. Consequently, we present systolic area as a useful device-derived marker for indicating change in contractile state in the setting of MCS support with comparisons to three widely accepted hemodynamically derived measures, ESPVR, PRSWi, and d*P*/d*t*_max_, that are all normalized to measure relative changes. Due to the focus of systolic area on only the contractile phase of the cardiac cycle, it is expected that d*P*/d*t*_max_ is the ideal reference measure to evaluate systolic area. However, comparisons between ESPVR and PRSWi when available serve as a useful benchmark to evaluate and reference.

Animal models with research-grade measurements of pressure–volume loops during known interventions enabled multiple avenues of validation. ESPVR and PRSWi were calculated using pressure–volume measurement as well as d*P*/d*t*_max_. At a qualitative level, all metrics remained consistent with expected responses for each intervention (Fig. 3A, B). Across all animals, correlation between normalized metrics remained similar with no significant differences in performance when compared with the other metrics (Fig. 4A). Results with patient data also showed ability to track changes in contractility in a time-series (Fig. 3C) and good correlation (Fig. 4B), though only d*P*/d*t*_max_ was available due to clinical limitations.

Notably, all contractility measures remained similarly correlated but not calibrated, even between the existing contractility markers. However, this does not prevent this approach from being an indicator of changing contractility. This diagnostic capability is evaluated using ROC analysis (Fig. 5). Directional changes in contractility as determined by moving average changepoint detection in d*P*/d*t*_max_ were compared with the results from systolic area with similar results between animal (AUC = 0.92) and patient datasets (AUC 0.90). Furthermore, there was no significant deviation in performance between increasing (AUC 0.99) and decreasing (AUC 0.95) contractility. As a comparison, these results were comparable or superior to results from ESPVR (AUC 0.89) and PRSWi (AUC 0.80) compared to d*P*/d*t*_max_. This indicates that changes in systolic area can in itself be readily used as a useful predictor for increasing or decreasing contractility. While a calibrated relationship between systolic area and each metric may be found with device-specific characterization, these results show that changes in contractility and trends can be adequately captured from existing MCS devices without alteration.

### Considerations and future work

Device hysteresis can be used to provide insights into changing physiologic state. Here, we demonstrate its utility via the hysteresis-derived systolic area as a new measure of contractility that can be calculated in real-time without additional intervention in the setting of MCS use. This measure can continuously monitor trends in contractility during MCS support and may be readily implemented for the care of patients in cardiogenic shock in order to provide immediate feedback, especially during ramp tests and weaning protocols. Indeed, we investigated the approach in patients undergoing PCI, and future steps include implementation during longer duration MCS use within intensive care units and in support of cardiogenic shock. This approach allows continuous monitoring and provides immediate feedback during ramp tests and weaning protocols. In addition, future investigation of device-specific characterization methods could lead to calibrated absolute measures of hysteresis and contractility rather than normalized trends. Another limitation of this work is the reliance on the Impella-specific design and device signals. While the concept may be generalized to other forms of percutaneous MCS, the approach as described here is reliant on signals that are readily available and specific to the Impella design.

Widely implementing this measure for MCS devices in ICUs will also lead to increased data that can contribute to determining optimal control of these devices towards improved outcomes. Future studies with contractility trends stratified by patient outcomes can determine trajectories and support strategies that lead to recovery. These data are critical to informing future automated control of these devices and prognostic capabilities.

Together with our other work to estimate end-diastolic pressure and cardiac output [14–16], this MCS device-derived contractility parameter will be a critical indicator of underlying contractile state to guide titration, prompt further intervention, and allow future automated closed-loop control of percutaneous MCS devices. Real-time contractility quantification during the entire duration of mechanical circulatory support will make clinical practice safer and more efficient by indicating alterations in patient state that prompt changes in therapy or further diagnostic investigation with other devices and imaging.

## Conclusions

Real-time quantification of cardiac state during the entire duration of mechanical circulatory support will make clinical practice safer and more efficient. Here, we employ a hysteresis estimation technique to indicate alterations in native contractility that can prompt changes in therapy or further diagnostic investigation with other devices and imaging. Future studies with contractility trends stratified by patient outcomes can determine trajectories and support strategies that lead to recovery. These data are critical to informing future automated control of these devices and prognostic capabilities.

## Data Availability

The datasets used and/or analyzed during the current study are available from the corresponding author on reasonable request.

## Abbreviations

MCS: Mechanical circulatory support
ESPVR: End-systolic pressure–volume relationship
PRSWi: Preload recruitable stroke work index
d*P*/d*t*_max_: Maximum slope for left ventricular pressure
*V*_0_: End-systolic pressure–volume relationship volume intercept
LVP: Left ventricular pressure
